# Immunity to vaccine-preventable diseases among paediatric healthcare workers in Denmark, 2019

**DOI:** 10.2807/1560-7917.ES.2021.26.17.2001167

**Published:** 2021-04-29

**Authors:** Marie-Louise von Linstow, Alex Yde Nielsen, Nikolai Kirkby, Anna Eltvedt, Thilde Nordmann Winther, Allan Bybeck Nielsen, Didi Bang, Anja Poulsen

**Affiliations:** 1 Department of Paediatrics and Adolescent Medicine, The Juliane Marie Centre, Copenhagen University Hospital Rigshospitalet, Copenhagen, Denmark; 2 Department of Clinical Microbiology, Copenhagen University Hospital Rigshospitalet, Copenhagen, Denmark; 3 Department of Paediatrics and Adolescent Medicine, Nordsjællands Hospital, Hillerød, Denmark; 4 Department of Paediatrics and Adolescent Medicine, Copenhagen University Hospital Hvidovre, Hvidovre, Denmark; 5 Virus and Microbiological Special Diagnostics, Infectious Disease Preparedness, Statens Serum Institut, Copenhagen, Denmark

**Keywords:** Antibodies, healthcare workers, vaccination, immunity, vaccine-preventable diseases, seroprevalence

## Abstract

**Background:**

Healthcare workers (HCW) have been identified as index cases in disease outbreaks of vaccine-preventable diseases (VPD) in hospitals.

**Aim:**

We investigated whether Danish paediatric HCW were protected against selected serious VPD.

**Methods:**

We included 90% of staff members from two paediatric departments. All 555 HCW (496 women) supplied a blood sample for serology and filled in a questionnaire. Antibodies were measured with enzyme immunoassay against measles, mumps, rubella (MMR), varicella zoster, pertussis toxin and diphtheria toxin.

**Results:**

Protective levels of IgG were found for measles (90.3%), mumps (86.5%), rubella (92.3%), varicella (98.6%) and diphtheria (80.5%). We found seropositivity for all three MMR components in 421 (75.9%) HCW, lowest in those younger than 36 years (63.3%). Only 28 (5%) HCW had measurable IgG to pertussis. HCW with self-reported immunity defined as previous infection or vaccination, had protective levels of IgG against measles, mumps, rubella and varicella in 87.4–98.8% of cases, not significantly higher than in those not reporting immunity. Previous history of disease had a high positive predictive value (PPV) of 96.8–98.8%. The PPV for previous vaccination ranged from 82.5% to 90.3%. In contrast, negative predictive values of self-reported history of disease and vaccination were remarkably low for all diseases.

**Conclusion:**

The immunity gaps found primarily in young HCW indicate a need for a screening and vaccination strategy for this group. Considering the poor correlation between self-reported immunity and seropositivity, efforts should be made to check HCW’s immune status in order to identify those who would benefit from vaccination.

## Introduction

Measles, mumps, rubella, varicella, diphtheria and pertussis are highly contagious infectious diseases that can lead to fatal illness. Vaccines for these diseases have been a part of the childhood immunisation programme in Denmark for decades. The diphtheria–tetanus vaccine was introduced in 1950, replaced by the diphtheria–tetanus–pertussis (whole cell) vaccine in 1961, and the diphtheria–tetanus–pertussis–inactivated polio vaccine (DTaP-IPV) in 1997. The measles–mumps–rubella (MMR) vaccine was included in 1987. Two varicella zoster vaccines (Varivax and Varilrix) are available in Denmark, however not as part of the national vaccination programme. Most of the Danish population acquire chickenpox during childhood and the seronegative status in adults is unknown. Despite acceptable vaccination coverage in Denmark (> 90% in recent years for both the first dose of MMR given at 15 months of age and for the second MMR dose given at age 4 years), several cases of measles, often imported from other European countries, have recently been recorded [1].

The MMR vaccine is considered highly effective, but suboptimal long-term immunity and seronegativity in immunised individuals after two doses of MMR vaccine have been reported [2,3]. Studies in healthcare workers (HCW) from Europe, Japan and Australia found that 6–19% were seronegative to measles, 6–32% were seronegative to mumps and 3–22% were seronegative to rubella [4-7]. Paediatric HCW are at particular risk of work-related exposure and transmission of these previously common childhood diseases, and several countries have reported nosocomial outbreaks of measles, mumps, pertussis and varicella, involving both patients and HCW [8-13].

Pertussis can be life-threatening for infants, and the DTaP vaccine only provides limited protection with vaccine efficacy of 40–85% and a decay over time of antibodies against vaccine proteins [14]. In recent years, 800–2,000 pertussis cases (15–35 per 100,000) have been diagnosed annually in Denmark, with the largest incidence among children younger than 1 year. In 2019, the annual incidence increased to 64 per 100,000, and this national epidemic continued until the coronavirus disease (COVID-19) lockdown in mid-March 2020.

Many European countries such as Austria, Finland and Italy recommend vaccination of all HCW, while others like Norway and the United Kingdom recommend vaccination of specific groups, such as paediatricians and neonatal staff [15]. Mandatory vaccination against e.g. pertussis, measles, mumps, rubella and diphtheria has been implemented in Albania, Croatia, Portugal and Slovenia as well as in parts of the United States [15,16].

In this study, we explored self-reported immunity in paediatric HCW and seroprevalence against the following vaccine-preventable diseases (VPD): measles, mumps, rubella, varicella zoster, pertussis and diphtheria. The study was performed in Denmark, a country that does not have national recommendations for vaccination of HCW, except for hepatitis B in specific groups.

## Methods

### Study design and population

From May to August 2019, all HCW employed at two paediatric departments in the greater area of Copenhagen were invited to participate in the project. We defined HCW as nurses, physicians, medical and nursing students, secretaries, dieticians, clowns, cleaning staff and others with direct or indirect contact with patients or access to patient rooms. The departments included were the Departments of Paediatrics and Adolescent Medicine, Rigshospitalet (tertiary care centre) and Hillerød Hospital (general hospital). To avoid including subjects who had not yet seroconverted or who could have received antibodies through transfusions, HCW who were given vaccinations, blood products or immunoglobulins during the previous 2 months were excluded from participation. HCW with recent infection were included in the study.

### Data collection

HCW who volunteered to participate in the study provided a blood sample for serology and filled in a structured questionnaire. The questionnaire collected demographic data including history of infection or vaccination against the following VPD: measles, mumps, rubella, varicella, pertussis and diphtheria. Data were self-reported and registered anonymously. Information was based on recollection and vaccination cards, if available. Self-reported immunity against measles, mumps, rubella and varicella was defined as previous infection or vaccination against the disease. Non-immunity was defined as no history or no knowledge of previous infection or vaccination. Pertussis and diphtheria were not included in these definitions because immunity in these cases wanes after disease or vaccination.

### Laboratory analysis

Blood samples were stored at −80 °C until analysis. Sera were tested for IgG antibodies specific for measles, mumps, rubella and varicella zoster viruses, and *Bordetella pertussis* toxin at the Department of Clinical Microbiology, Rigshospitalet, Copenhagen using DiaSorin Liaison chemiluminescence immunoassay (DiaSorin S.p.A., Saluggia, Italy). The quantitative cut-off value for seronegativity was < 13.5 arbitrary units (AU)/mL for measles, < 9.0 AU/mL for mumps, < 7.0 international units (IU)/mL for rubella and < 50 mIU/mL for varicella. The seropositivity cut-off was ≥ 16.5 AU/mL for measles, ≥ 11.0 AU/mL for mumps, ≥ 10 IU/mL for rubella and ≥ 100 mIU/mL for varicella. Titres in between were defined as equivocal. For pertussis, a titre ≥ 40 IU/mL was defined as indicative of recent contact with *B. pertussis* [17].

Samples with equivocal and negative serological results for measles, mumps, rubella or varicella using the Liaison assay were reassessed with an enzyme-linked fluorescent assay (VIDAS – BioMérieux, Marcy l'Étoile, France) at the Department of Clinical Microbiology, Hvidovre University Hospital. The test value for each sample was obtained by calculating the ratio of the relative fluorescence value of each sample to a standard. The quantitative cut-off value for seronegativity was < 0.5 for measles, < 0.35 for mumps and < 0.6 for varicella. The seropositivity cut-off was ≥ 0.7 for measles, ≥ 0.5 for mumps and ≥ 0.9 for varicella. IgG values specific for rubella virus were calculated as IU/mL considering values < 10 IU/mL to be negative and values ≥ 15 IU/mL to be positive. Titres in between were defined as equivocal.

Sera tested positive in one of the test systems were considered positive. Equivocal test results were counted as negative for measles, mumps and rubella and positive for varicella as agreed by the European sero-epidemiology network [18].

IgG against *Corynebacterium diphtheriae* toxin was analysed by enzyme-linked immunosorbent assay at Statens Serum Institute, Copenhagen. Titres > 0.1 kIU/L were recorded as protective, 0.01–0.1 kIU/L as limited protective and < 0.01 kIU/L as not protective.

HCW who were seronegative to measles, mumps, rubella, varicella or diphtheria were offered healthcare-provided vaccination in the department. HCW with negative pertussis IgG were informed of the possibility of receiving the DTaP vaccine from their general practitioner as it was not offered free of charge at the hospital.

### Statistical analysis

Categorical variables were compared with chi-squared tests or Fisher’s exact test, considering a value of p < 0.05 statistically significant. Logistic regression analysis was applied to investigate factors associated with seronegativity. The following independent variables were assessed in the univariate analysis: sex, age, profession and history of previous vaccination or disease. Variables with a significance level of p < 0.1 in the univariate analysis were entered in the multiple regression model (forward selection). Missing data were left out of the analysis. Positive predictive values (PPV) and negative predictive values (NPV) were calculated for those who remembered their history for disease and vaccination, using the serological test result as gold standard. Subjects answering ‘unsure’ to a history of disease or vaccination were excluded from analysis. Data were analysed using the SPSS software, version 25 for Windows.

### Ethical statement

Written consent was obtained from all participants. The study was approved by the Ethics Committee of the Capital Region of Denmark (H-18057042) and the Data Protection Agency (VD-2019–122).

## Results

A total of 555 (90%) of 617 HCW employed at the two paediatric departments were included in the study; 303 nurses, 131 physicians, 40 students, 17 dieticians or physiotherapists and 64 non-clinical personnel. Most of the participants, 496 of 555 (89.4%) were women and 49 (8.8%) were younger than 26 years, 158 (28.5%) were 26–35 years, 142 (25.6%) were 36–45 years, 110 (19.8%) were 46–55 years, 78 (14.1%) were 56–65 years, and 18 (3.2%) were older than 65 years. Non-participants included 57 (91.9%) women and 40 (64.5%) were nurses. Further characteristics of participants are available in a recent publication focusing on opinions on vaccination of hospital personnel [19].

### Self-reported immunity

The HCW’s self-reported vaccination status and disease history are shown in Table 1.

**Table 1:** Self-reported history of disease and vaccination in paediatric healthcare workers, Denmark, 2019 (n = 555)

	Disease							Vaccination						
	Total^a^	Yes		No		Unsure		Total^a^	Yes		No		Unsure	
	n	n	%	n	%	n	%	n	n	%	n	%	n	%
Measles	552	203	36.8	228	41.3	121	21.9	543	267	49.2	155	28.5	121	22.3
Mumps	549	148	27.0	245	44.6	156	28.4	540	257	47.6	149	27.6	134	24.8
Rubella	551	186	33.8	222	40.3	143	26.0	543	278	51.2	145	26.7	120	22.1
Varicella	553	481	87.0	13	2.4	59	10.7	531	14	2.6	392	73.8	125	23.5
Diphtheria	550	5	0.9	369	67.1	176	32.0	548	372	67.9	25	4.6	151	27.6
Pertussis	550	62	11.3	263	47.8	225	40.9	544	304	55.9	68	12.5	172	31.6

^a^ Total with available information.

The percentage of HCW reporting previous disease was high for varicella (87.0%) compared with all other diseases. A history of vaccination was most common for diphtheria (67.9%) compared with approximately half of HCW for MMR and only 2.6% for varicella zoster. Five HCW claimed to have had diphtheria, of whom two were also vaccinated. These were all above 45 years of age.

Between 22.1% and 31.6% of HCW were unsure of their vaccination status depending on disease. Similarly, a high proportion of HCW did not know if they had previously been infected, ranging from 10.7% for varicella to 40.9% for pertussis. HCW who recalled both their vaccination status and disease history were 361 of 540 (66.9%) for measles, 322 of 535 (60.2%) for mumps, 342 of 539 (63.5%) for rubella, 378 of 530 (71.3%) for varicella, 309 of 543 (56.9%) for diphtheria and 257 of 539 (47.7%) for pertussis.

Self-reported immunity defined as previous vaccination or disease was reported in 441 of 552 (79.9%) for measles, 381 of 546 (69.8%) for mumps, 433 of 551 (78.6%) for rubella and 486 of 553 (87.9%) for varicella, leaving 12.1–30.2% self-reported non-immune. Based on recollection, 238 (42.9%) of all included HCW were potentially susceptible to one or more VPD.

### Seroprevalence of antibodies

Protective levels of IgG were detected for measles, mumps and rubella in 501 (90.3%), 480 (86.5%) and 512 (92.3%) HCW, respectively (Table 1). HCW younger than 36 years had the lowest seroprevalences. Most seropositive sera were identified with the Liaison analyser; however, an additional 38, 88 and 67 sera were found IgG-positive for, respectively, measles, mumps and rubella using the VIDAS robot.

Seropositivity for all three MMR components was found in 421 (75.9%) HCW, depending on age; the oldest age group of 66–75 years had 100% seropositivity compared with 131 of 207 (63.3%) HCW under the age of 36 years. Four HCW did not have detectable antibody levels to any of the measles, mumps or rubella viruses and 130 (23.4%) lacked IgG to one or two components only. Thirty-two of 311 (10.3%) women under the age of 45 years and therefore potentially fertile were seronegative for rubella. Of these, 25 reported previous vaccination and three reported previous rubella infection.

Protective IgG levels for varicella zoster virus were found in 547 (98.6%) HCW, of whom 520 were detected by the Liaison assay and an additional 27 by the VIDAS method. Six of the eight seronegative HCW recalled previous varicella infection. Seven HCW denied previous varicella infection and vaccination, all of whom were seropositive. An additional 60 HCW were unsure if they ever had varicella or the vaccine, and 58 of them were seropositive. There were no significant differences between age groups.

**Figure 1:**
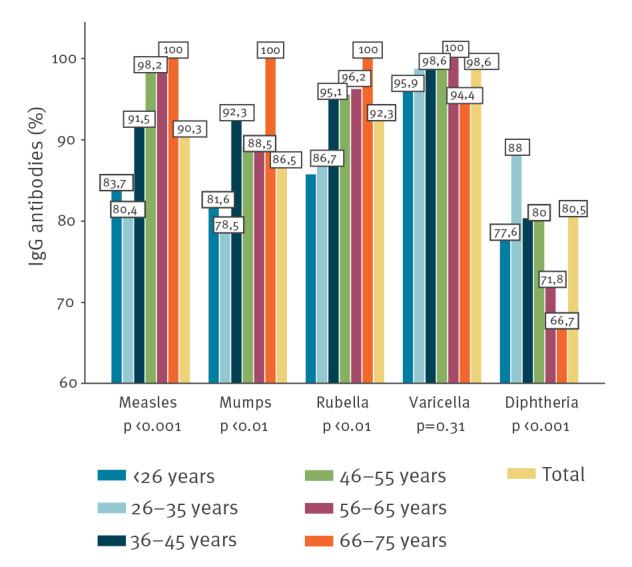
Seropositivity against vaccine-preventable diseases in paediatric healthcare workers, by age, Denmark, 2019 (n = 555)

Diphtheria antibodies > 0.1 IU/mL were present in 447 of 555 (80.5%) HCW. Most of the remaining HCW (n = 105) had antibody levels 0.1–0.01 IU/mL indicating partial protection and only three had levels < 0.01 IU/mL. Seroprevalence was lowest in the oldest age group with only 66.7% considered protected (Figure 1).

Altogether, 222 (40%) of the HCW had suboptimal IgG levels towards at least one of the antigens tested: measles, mumps, rubella, varicella or diphtheria. Physicians had the lowest seronegativity of 26.7% compared with 42.3% of nurses and 48.8% of other professions (p < 0.01). There was no difference according to sex or between hospitals.

One HCW had anti-pertussis-IgG titre > 100 IU/mL. This employee was vaccinated in 2015 and had no recollection of a previous pertussis infection. Twenty-seven HCW had intermediate titre levels between 40 and 100 IU/mL, four of them recalled previous pertussis infection and 15 reported previous vaccination, however not within the past year. The 27 HCW represented most age groups and 16 were nurses. The remaining 527 HCW had IgG levels < 40 IU/mL.

### Self-reported immunity and seroprevalence

Comparing information of self-reported history of disease or vaccination with serology results, HCW answering ‘yes’ to a history of disease but ‘no’ to history of vaccination had the highest seropositivity to measles, mumps, rubella and varicella (97.4% for rubella, 99.4% for varicella) followed by HCW reporting a history of both disease and vaccination (Table 2).

**Table 2:** Self-reported history of disease and vaccination and serology results for measles, mumps, rubella and varicella in paediatric healthcare workers, Denmark, 2019 (n = 555)

EIA result	Measles n = 361		Mumps n = 322		Rubella n = 342		Varicella n = 378	
	n	%	n	%	n	%	n	%
HD+ , HV−								
Positive	132	98.5	91	98.9	113	97.4	358	99.4
Negative	2	1.5	1	1.1	3	2.6	2	0.6
Total	134		92		116		360	
HD−, HV+								
Positive	163	85.3	141	78.3	169	89.9	2	100
Negative	28	14.7	39	21.7	19	10.1	0	0
Total	191		180		188		2	
HD+ , HV+								
Positive	28	96.6	23	95.8	29	93.5	7	77.8
Negative	1	3.4	1	4.2	2	6.5	2	22.2
Total	29		24		31		9	
HD−, HV−								
Positive	7	100	20	76.9	7	100	7	100
Negative	0	0	6	23.1	0	0	0	0
Total	7		26		7		7	

EIA: enzyme Immunoassay; HD: history of disease; HV: history of vaccination.

Subjects who had unknown vaccination or disease history were excluded from analysis.

**Figure 2:**
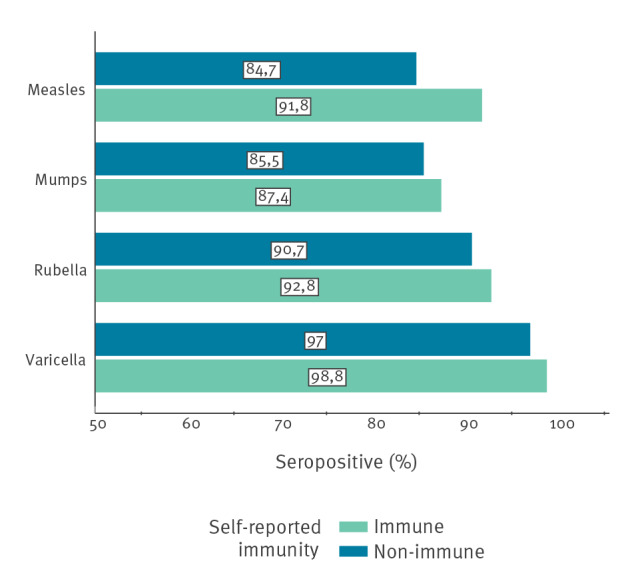
Seroprevalence to measles, mumps, rubella and varicella in paediatric healthcare workers, by self-reported immunity, Denmark, 2019 (n = 555)

Vaccinated HCW who had no history of disease had seroprevalences ranging from 78.3% for mumps to 100% for varicella. HCW who reported neither a history of disease nor a history of vaccination showed seropositivity rates of 100% for measles, rubella and varicella, and of 76.9% for mumps. Including subjects who were unsure of previous disease or vaccination as non-immune, seroprevalences for self-reported immune vs non-immune HCW were very similar (Figure 2).

Non-immune defined as no or unknown history of disease and vaccination.

p < 0.05 for measles.

The odds of being seronegative were higher for HCW under the age of 36 years compared with those older than 55 years, with odds ratios of 22 (95% confidence interval (CI): 2.98–163.09) for measles, 2.5 (95% CI: 1.18–5.44) for mumps and 4.9 (95% CI: 1.44–16.37) for rubella, a finding that remained significant in the multivariate analysis for measles and rubella (Tables 3 and 4). Compared with physicians, the odds ratio for negative diphtheria toxin IgG in multivariate analysis was 2.7 (95% CI: 1.38–5.20) for other HCW (except nurses). Not having a history of previous disease showed odds ratios of 12.5 (95% CI: 3.79–41.27) for measles, 9.5 (95% CI: 3.34–26.78) for mumps and 3.8 (95% CI: 1.53–9.49) for rubella seronegativity; this remained significant only for mumps in the multivariate model.

**Table 3:** Odds ratios of seronegativity to measles, mumps, rubella, diphtheria and varicella zoster in paediatric healthcare workers, univariate analysis, Denmark, 2019 (n = 555)

	Measles seronegativity		Mumps seronegativity		Rubella seronegativity		Diphtheria seronegativity		Varicella seronegativity	
	OR	95% CI	OR	95% CI	OR	95% CI	OR	95% CI	OR	95% CI
Age group (years)										
> 55	**1 (ref) **		**1 (ref)**		**1 (ref)**		**1 (ref)**		1 (ref)	
46–55	**1.76**	**0.16–19.71**	**1.18**	**0.48–2.94**	**1.48**	**0.34–6.35**	**0.61**	**0.32–1.15**	0.87	0.54–14.13
36–45	**8.77**	**1.12–68.60**	**0.81**	**0.32–2.04**	**1.61**	**0.41–6.38**	**0.60**	**0.33–1.10**	1.36	0.21–15.18
< 36	**22.05**	**2.98–163.09**	**2.54**	**1.18–5.44**	**4.85**	**1.44–16.37**	**0.41**	**0.23–0.74**	1.87	0.21–16.98
Profession										
Physician	1 (ref)		1 (ref)		**1 (ref)**		**1 (ref)**		1 (ref)	
Nurse	1.91	0.89–4.08	1.19	0.63–2.24	**3.14**	**1.08–9.16**	**1.57**	**0.88–2.83**	0.65	0.11–3.95
Other	0.94	0.35–2.51	1.49	0.72–3.06	**3.40**	**1.07–10.85**	**2.53**	**1.33–4.83**	1.60	0.26–9.74
Vaccinated										
Yes	**1 (ref)**		**1 (ref)**		**1 (ref)**		1 (ref)		**1 (ref)**	
No	**0.09**	**0.02–0.38**	**0.34**	**0.17–0.70**	**0.26**	**0.09–0.77**	1.46	0.56–3.81	**0.31**	**0.004–0.24**
Unsure	**1.12**	**0.60–2.10**	**0.64**	**0.37–1.18**	**0.94**	**0.45–1.96**	1.30	0.81–2.07	**0.19**	**0.03–1.20**
Previous disease										
Yes	**1 (ref)**		**1 (ref)**		**1 (ref)**		1 (ref)		**1 (ref)**	
No	**12.5**	**3.79–41.27**	**9.46**	**3.34–26.78**	**3.81**	**1.53–9.49**	0.86	0.09–7.78	**6.60**	**0.74–59.13**
Unsure	**9.43**	**2.67–33.32**	**4.99**	**1.66–15.05**	**2.75**	**1.01–7.51**	1.18	0.13–10.83	**1.37**	**0.16–11.54**

CI: confidence interval; OR: odds ratio.

Variables in bold were included in the multivariate analysis.

**Table 4:** Adjusted odds ratios of seronegativity to measles, mumps, rubella, diphtheria and varicella zoster in paediatric healthcare workers, multivariate analysis, Denmark, 2019 (n = 555)

	Measles seronegativity		Mumps seronegativity		Rubella seronegativity		Diphtheria seronegativity		Varicella seronegativity	
	aOR	95% CI	aOR	95% CI	aOR	95% CI	aOR	95% CI	aOR	95% CI
Age group (years)										
> 55	1 (ref)		NS		1 (ref)		1 (ref)		NI	
46–55	1.65	0.15–18.78			1.50	0.35–6.44	0.63	0.32–1.24		
36–45	5.77	0.68–48.91			1.52	0.38–6.04	0.68	0.37–1.27		
< 36	15.12	1.78–128.53			4.50	1.33–15.21	0.40	0.22–0.74		
Profession										
Physician	NI		NI		NS		1 (ref)		NI	
Nurse							1.78	0.97–3.27		
Other							2.68	1.38–5.20		
Vaccinated										
Yes	1 (ref)		NS		NS		NI		1 (ref)	
No	0.45	0.09–2.32							0.31	0.004–0.24
Unsure	1.99	1.00–3.97							0.20	0.03–1.21
Previous disease										
Yes	NS		1 (ref)		NS		NI		NS	
No			11.83	3.62–38.71						
Unsure			6.13	1.78–21.31						

aOR: adjusted odds ratio; CI: confidence interval; NI: not included in analysis; NS: not significant.

Self-reported history of measles, mumps, rubella or varicella had a high PPV ranging from 96.8% for rubella to 98.8% for varicella (Table 5). The PPV for vaccination ranged from 82.5% to 90.3%. In contrast, NPV of self-reported history of disease and vaccination were remarkably low for all diseases.

**Table 5:** Predictive value of self-reported history of disease and vaccination in paediatric healthcare workers, Denmark, 2019 (n = 555)

Disease		EIA positive	EIA negative	Total	PPV		NPV	
		n	n	n	%	n/total	%	n/total
Measles								
HD	Yes	200	3	203	98.5	200/203	15.8	36/228
	No	192	36	228				
HV	Yes	233	34	267	87.3	233/267	1.3	2/155
	No	153	2	155				
Mumps								
HD	Yes	144	4	148	97.3	144/148	20.8	51/245
	No	194	51	245				
HV	Yes	212	45	257	82.5	212/257	6.7	10/149
	No	139	10	149				
Rubella								
HD	Yes	180	6	186	96.8	180/186	11.3	25/222
	No	197	25	222				
HV	Yes	251	27	278	90.3	251/278	2.8	4/145
	No	141	4	145				
Varicella								
HD	Yes	475	6	481	98.8	475/481	7.7	1/13
	No	12	1	13				
HV	Yes	12	2	14	85.7	12/14	0.5	2/392
	No	390	2	392				

EIA: enzyme immunoassay; HD: history of disease; HV: history of vaccination; NPV: negative predictive value; PPV: positive predictive value.

Subjects answering ‘unsure’ to a history of disease or vaccination were excluded from analysis.

## Discussion

In this study, we examined the self-reported and serological immunity to important VPD among 555 paediatric HCW in Denmark. Overall, protective levels of IgG were high for VZV, low for diphtheria and close to 90% for measles, mumps and rubella. A quarter of the HCW did not have IgG against at least one of the MMR components, which is in accordance with findings from Finland, where a quarter (39/157) of paediatric nurses had inadequate immunity to measles, mumps or rubella [4]. Lack of immunity was highest in the youngest HCW, a finding consistent with other studies [5-7]. IgG antibodies after disease are often higher than after vaccination [3,20]. The low prevalence of e.g. measles in the Danish society may explain the lower antibody titres in the youngest age group as vaccine-induced antibodies need boosting by re-exposure to persist.

Mumps had the highest seronegativity rate of the MMR vaccine components in our study. The mumps component of the vaccine is ca 88% effective after two doses, and protection is estimated to last 10–15 years [16]. Outbreaks in highly vaccinated populations still occur as do outbreaks with non-vaccine strains [21]. In Denmark, a 10-fold increase of parotitis in primarily young adults was seen from 2003 to 2013 but has since declined [22].

As for mumps, rubella antibodies may wane after 12–15 years. However, rubella surveillance data do not indicate that congenital rubella is increasing among vaccinated persons and Denmark achieved rubella elimination status in 2020. Nevertheless, the finding of sero-negative younger women in our study is of concern and occupational health services should contribute to ensure that female HCW stay protected against this VPD.

Varicella immunity was high with only eight (1.4%) seronegative HCW. Almost all were immune because of previous infection which may explain why their seroprevalence was higher than that found in vaccinated individuals [7,20].

The immunological implication of low or missing antibody titres despite vaccination is unclear [23,24]. In 2011, the United States Advisory Committee on Immunization Practices (ACIP) stated that HCW who are seronegative to measles, mumps or rubella and who have two documented doses of MMR vaccine should not receive an additional dose of MMR vaccine as this is presumptive evidence of immunity [25]. Nonetheless, several recent reports describe outbreaks of measles and mumps at hospitals involving previously vaccinated HCW, indicating waning immunity after vaccination [8,21,26]. Importantly, it appears that seronegative individuals with documented vaccination may have reduced risk of severe symptoms and be less likely to transmit the virus compared with seronegative unvaccinated individuals [8,9,12,26].

For single-sample serology, various cut-off values for IgG against pertussis toxin have been proposed [17]. Defining titres > 40 IU/mL as indicative of recent contact with *B. pertussis*, 28 HCW in this study (5%) had recently been infected, where only four recalled symptoms. This is worrying as asymptomatic infection and transmission may occur. In Denmark, pertussis vaccination is given at 3, 5 and 12 months with a booster dose at 5 years. However, pertussis antibodies wane rapidly within years of the vaccination [14]. Some European countries offer booster doses of pertussis vaccine every 10 years for the whole population [27], while others offer regular HCW vaccination [15]. In Denmark, no such programme is implemented, however during the recent pertussis epidemic, pertussis vaccination was offered for pregnant women. As pertussis infections still exist among Danish paediatric HCW, a booster dose in this group to protect unvaccinated infants in the hospital setting seems reasonable.

Only three (0.5%) HCW had undetectable levels of diphtheria antibodies, while 20% were partly protected. The last case of diphtheria in Denmark was seen in 1998, therefore unprotected HCW do not pose a high risk for patients.

We found that a history of previous disease was a good predictor of seropositivity to measles, mumps, rubella and varicella. However, the NPV for history of previous disease or vaccination was very poor, which is also observed in other studies [28,29]. A large proportion of HCW were unsure of their own vaccination or disease history, which is of concern because paediatric HCW are often exposed to infectious diseases, and may pose a risk to themselves, colleagues and patients if they are not protected.

Even though the prevalence of seronegativity in our study may seem acceptable, a single nosocomial outbreak may have considerable economic implications. The cost related to a hospital outbreak of 10 measles cases in HCW in Germany was estimated at EUR 700,000, including deficit relating to suspension of the personnel without immunity, the decrease in patients during and in the weeks after the outbreak and serology tests and vaccinations [9]. The best way to prevent such hospital-based outbreaks is to ensure that all HCW are fully immunised. Because the NPV of self-reported immunity was low, the most cost-effective and accurate immunisation strategy among HCW seems to be pre-vaccination IgG screening [29,30]. For such an initiative to succeed, HCW should be educated about the importance and limitations of vaccines and the severe manifestations of the VPD.

In this study, MMR booster vaccination was offered to 134 (24.1%) HCW lacking immunity to one or more components of the vaccine based on serology results. If vaccination recommendation had been based on self-reported immunity, 203 (36.6%) HCW would have received an MMR booster vaccination, including 152 subjects seropositive to all MMR components and only 51 of the 134 seronegative HCW. Similarly, if based on self-reported immunity to varicella, 67 (12.1%) HCW would require vaccination compared with only two (0.4%) based on serology and no history of previous chickenpox.

A strength of this study is the high response rate of 90%. We are not aware of similar studies involving all HCW categories including non-clinical personnel, students and volunteers from both a tertiary care centre and a general hospital. This makes our findings more generalisable to other paediatric wards in countries with similar vaccination programmes. To increase sensitivity and avoid overestimating the number of unprotected HCW and subsequent unnecessary vaccination, we used two different IgG enzyme immunoassays for antibody testing. The reduction in the number of seronegative HCW by including the VIDAS method was larger than expected. This emphasises the importance of considering more than one test method for seroprevalence studies.

This study has some limitations. Firstly, only 10.6% of participants were male and it is therefore difficult to generalise our findings to sex. Secondly, information about previous disease and vaccination was self-reported based on memory and less frequently on vaccination cards or health files. A mandatory electronic vaccination registry was implemented in 2015, but vaccinations given before this period are not all electronic and some respondents could not find proof of previous vaccinations. Opinions among our cohort on vaccination of HCW including influenza are covered elsewhere [19].

This study relied on measurements of humoral immunity to detect host disease susceptibility. A larger number than registered in this study may have been identified as immune if plaque reduction neutralisation (RPN) antibody assays or measurements of cell-mediated immunity had been added [24,31]. However, the RPN test is not suitable for larger seroprevalence studies because of technical and practical challenges.

## Conclusion

The immunity status of paediatric HCW from two Danish hospitals was in line with findings from other parts of the world. Immunity gaps were found primarily in young HCW, indicating a need for a screening and vaccination strategy for this group to protect them and to support the elimination goals for measles and rubella. The poor correlation between self-reported immunity and seropositivity emphasises a need for improving vaccine databases and efforts should be made to check immunity status of currently employed HCW to identify those who may benefit from a booster vaccination. Protection of HCW from VPD and reducing the magnitude of future hospital outbreaks calls for national guidelines on vaccination of HCW in Denmark and other countries that have not yet implemented such a strategy.
